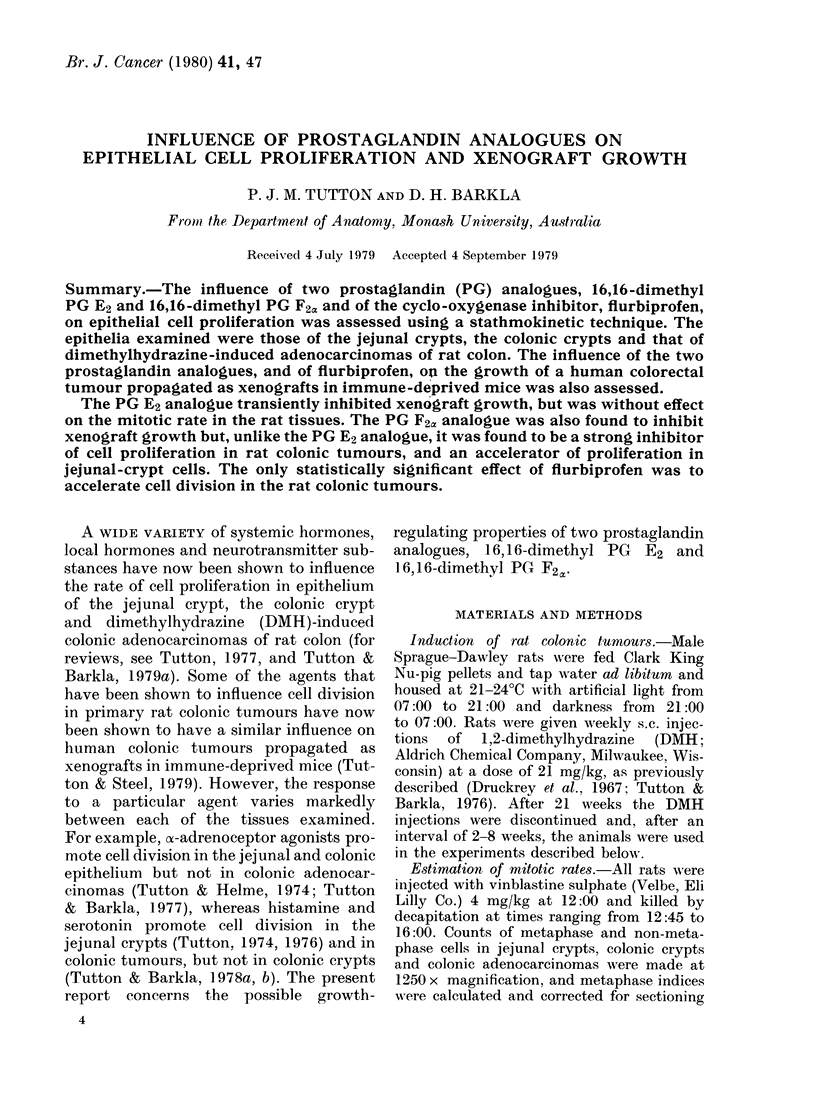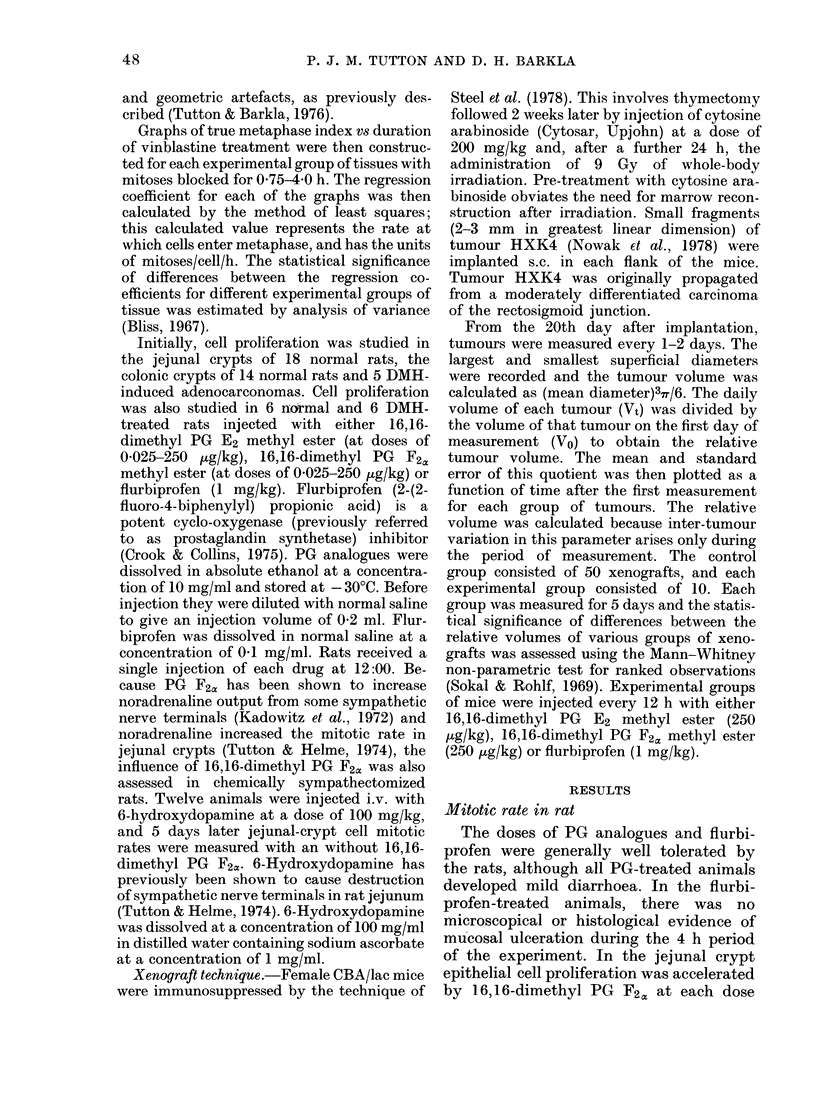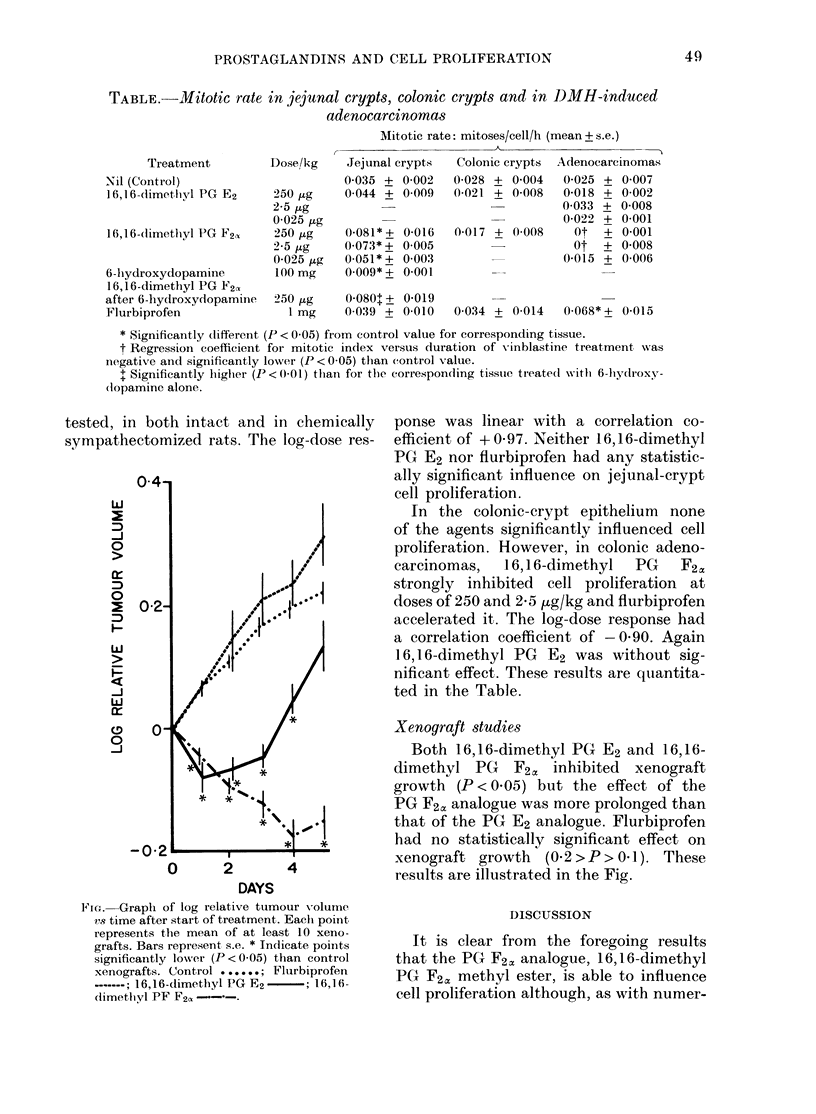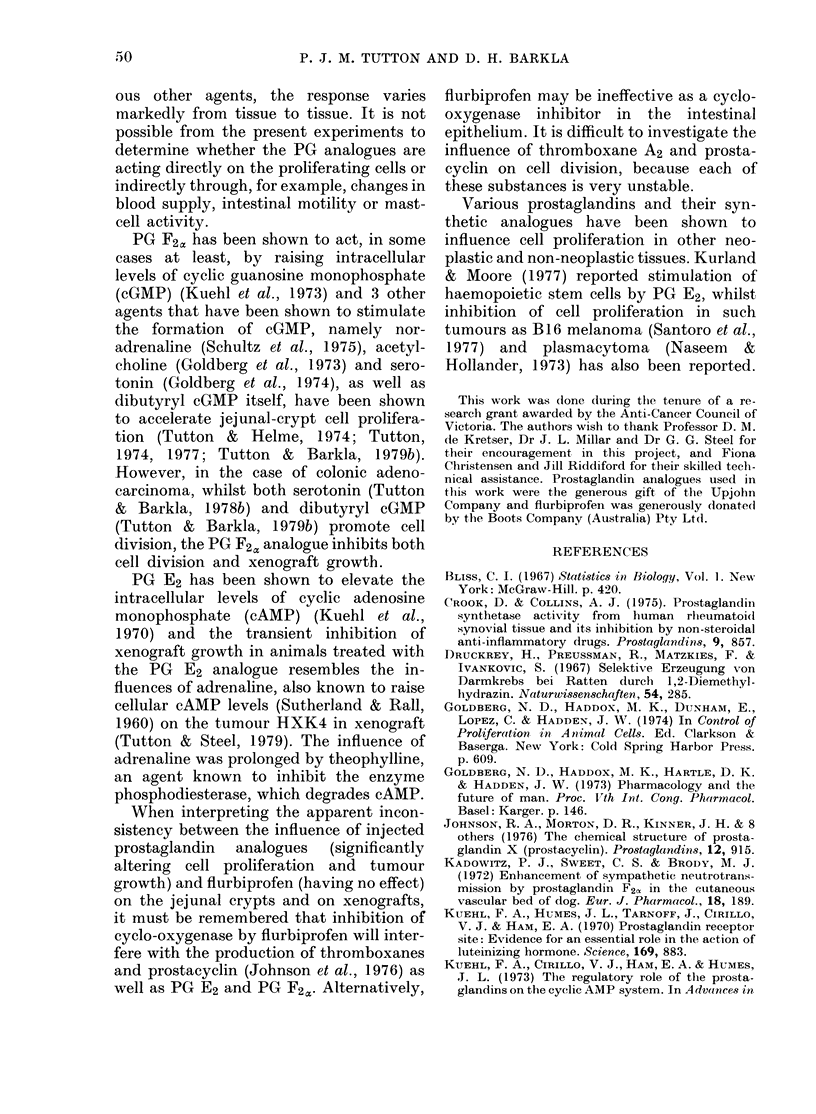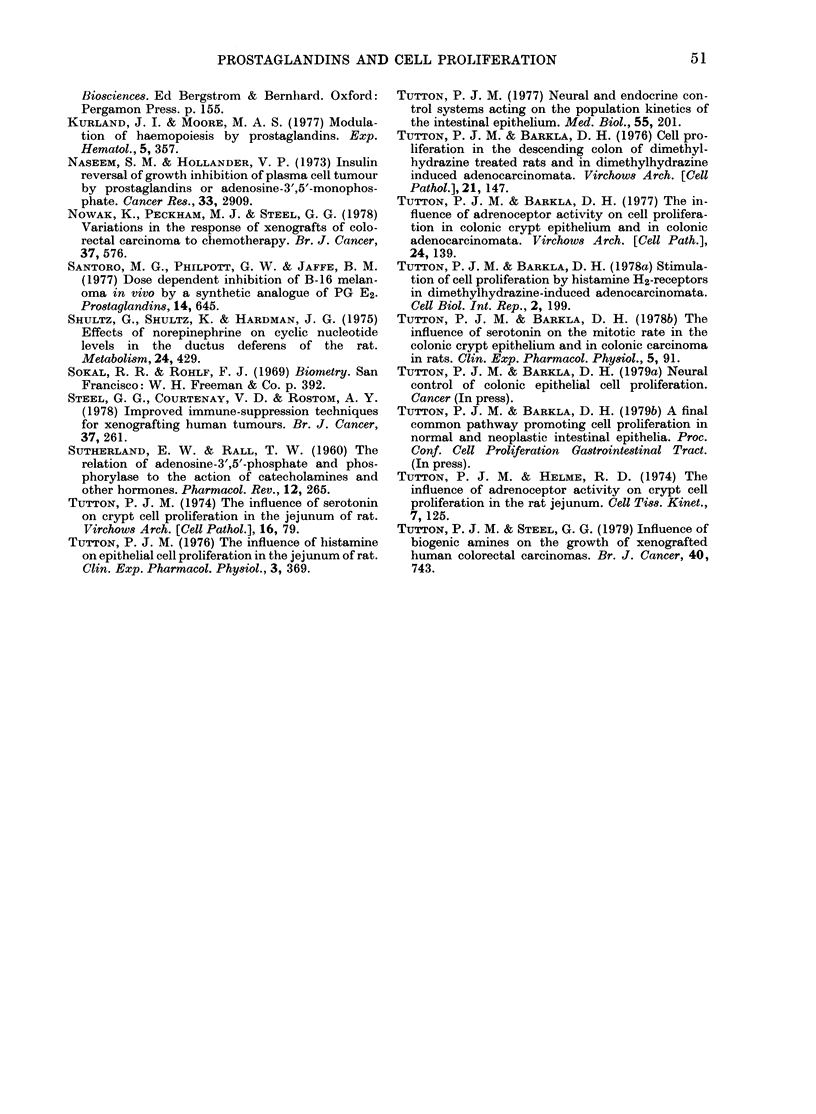# Influence of prostaglandin analogues on epithelial cell proliferation and xenograft growth.

**DOI:** 10.1038/bjc.1980.6

**Published:** 1980-01

**Authors:** P. J. Tutton, D. H. Barkla

## Abstract

The influence of two prostaglandin (PG) analogues, 16,16-dimethyl PG E2 and 16,16-dimethyl PG F2 alpha and of the cyclo-oxygenase inhibitor, flurbiprofen, on epithelial cell proliferation was assessed using a stathmokinetic technique. The epithelia examined were those of the jejunal crypts, the colonic crypts and that of dimethylhydrazine-induced adenocarcinomas of rat colon. The influence of the two prostaglandin analogues, and of flurbiprofen, on the growth of a human colorectal tumour propagated as xenografts in immune-deprived mice was also assessed. The PG E2 analogue transiently inhibited xenograft growth, but was without effect on the mitotic rate in the rat tissues. The PG F2 alpha analogue was also found to inhibit xenograft growth but, unlike the PG E2 analogue, it was found to be a strong inhibitor of cell proliferation in rat colonic tumours, and an accelerator of proliferation in jejunal-crypt cells. The only statistically significant effect of flurbiprofen was to accelerate cell division in the rat colonic tumours.


*Br. J. Cancer* (1980) **41**, 47

### INFLUENCE OF PROSTAGLANDIN ANALOGUES ON EPITHELIAL CELL PROLIFERATION AND XENOGRAFT GROWTH

P. J. M. TUTTON AND D. H. BARKLA

*From the Department of Anatomy, Monash University, Australia*

Received 4 July 1979 Accepted 4 September 1979

**Summary.—The influence of two prostaglandin (PG) analogues, 16,16-dimethyl PG $E_2$ and 16,16-dimethyl PG $F_{2\alpha}$ and of the cyclo-oxygenase inhibitor, flurbiprofen, on epithelial cell proliferation was assessed using a stathmokinetic technique. The epithelia examined were those of the jejunal crypts, the colonic crypts and that of dimethylhydrazine-induced adenocarcinomas of rat colon. The influence of the two prostaglandin analogues, and of flurbiprofen, on the growth of a human colorectal tumour propagated as xenografts in immune-deprived mice was also assessed.**

**The PG $E_2$ analogue transiently inhibited xenograft growth, but was without effect on the mitotic rate in the rat tissues. The PG $F_{2\alpha}$ analogue was also found to inhibit xenograft growth but, unlike the PG $E_2$ analogue, it was found to be a strong inhibitor of cell proliferation in rat colonic tumours, and an accelerator of proliferation in jejunal-crypt cells. The only statistically significant effect of flurbiprofen was to accelerate cell division in the rat colonic tumours.**

A WIDE VARIETY of systemic hormones, local hormones and neurotransmitter substances have now been shown to influence the rate of cell proliferation in epithelium of the jejunal crypt, the colonic crypt and dimethylhydrazine (DMH)-induced colonic adenocarcinomas of rat colon (for reviews, see Tutton, 1977, and Tutton & Barkla, 1979*a*). Some of the agents that have been shown to influence cell division in primary rat colonic tumours have now been shown to have a similar influence on human colonic tumours propagated as xenografts in immune-deprived mice (Tutton & Steel, 1979). However, the response to a particular agent varies markedly between each of the tissues examined. For example, $\alpha$-adrenoceptor agonists promote cell division in the jejunal and colonic epithelium but not in colonic adenocarcinomas (Tutton & Helme, 1974; Tutton & Barkla, 1977), whereas histamine and serotonin promote cell division in the jejunal crypts (Tutton, 1974, 1976) and in colonic tumours, but not in colonic crypts (Tutton & Barkla, 1978*a*, *b*). The present report concerns the possible growth-regulating properties of two prostaglandin analogues, 16,16-dimethyl PG $E_2$ and 16,16-dimethyl PG $F_{2\alpha}$.

### MATERIALS AND METHODS

*Induction of rat colonic tumours.*—Male Sprague–Dawley rats were fed Clark King Nu-pig pellets and tap water *ad libitum* and housed at 21–24°C with artificial light from 07:00 to 21:00 and darkness from 21:00 to 07:00. Rats were given weekly s.c. injections of 1,2-dimethylhydrazine (DMH; Aldrich Chemical Company, Milwaukee, Wisconsin) at a dose of 21 mg/kg, as previously described (Druckrey *et al.*, 1967; Tutton & Barkla, 1976). After 21 weeks the DMH injections were discontinued and, after an interval of 2–8 weeks, the animals were used in the experiments described below.

*Estimation of mitotic rates.*—All rats were injected with vinblastine sulphate (Velbe, Eli Lilly Co.) 4 mg/kg at 12:00 and killed by decapitation at times ranging from 12:45 to 16:00. Counts of metaphase and non-metaphase cells in jejunal crypts, colonic crypts and colonic adenocarcinomas were made at 1250× magnification, and metaphase indices were calculated and corrected for sectioning and geometric artefacts, as previously described (Tutton & Barkla, 1976).

Graphs of true metaphase index *vs* duration of vinblastine treatment were then constructed for each experimental group of tissues with mitoses blocked for 0·75–4·0 h. The regression coefficient for each of the graphs was then calculated by the method of least squares; this calculated value represents the rate at which cells enter metaphase, and has the units of mitoses/cell/h. The statistical significance of differences between the regression coefficients for different experimental groups of tissue was estimated by analysis of variance (Bliss, 1967).

Initially, cell proliferation was studied in the jejunal crypts of 18 normal rats, the colonic crypts of 14 normal rats and 5 DMH-induced adenocarconomas. Cell proliferation was also studied in 6 normal and 6 DMH-treated rats injected with either 16,16-dimethyl PG $E_2$ methyl ester (at doses of 0·025–250 $\mu$g/kg), 16,16-dimethyl PG $F_{2\alpha}$ methyl ester (at doses of 0·025–250 $\mu$g/kg) or flurbiprofen (1 mg/kg). Flurbiprofen (2-(2-fluoro-4-biphenylyl) propionic acid) is a potent cyclo-oxygenase (previously referred to as prostaglandin synthetase) inhibitor (Crook & Collins, 1975). PG analogues were dissolved in absolute ethanol at a concentration of 10 mg/ml and stored at −30°C. Before injection they were diluted with normal saline to give an injection volume of 0·2 ml. Flurbiprofen was dissolved in normal saline at a concentration of 0·1 mg/ml. Rats received a single injection of each drug at 12:00. Because PG $F_{2\alpha}$ has been shown to increase noradrenaline output from some sympathetic nerve terminals (Kadowitz *et al.*, 1972) and noradrenaline increased the mitotic rate in jejunal crypts (Tutton & Helme, 1974), the influence of 16,16-dimethyl PG $F_{2\alpha}$ was also assessed in chemically sympathectomized rats. Twelve animals were injected i.v. with 6-hydroxydopamine at a dose of 100 mg/kg, and 5 days later jejunal-crypt cell mitotic rates were measured with an without 16,16-dimethyl PG $F_{2\alpha}$. 6-Hydroxydopamine has previously been shown to cause destruction of sympathetic nerve terminals in rat jejunum (Tutton & Helme, 1974). 6-Hydroxydopamine was dissolved at a concentration of 100 mg/ml in distilled water containing sodium ascorbate at a concentration of 1 mg/ml.

*Xenograft technique.*—Female CBA/lac mice were immunosuppressed by the technique of Steel *et al.* (1978). This involves thymectomy followed 2 weeks later by injection of cytosine arabinoside (Cytosar, Upjohn) at a dose of 200 mg/kg and, after a further 24 h, the administration of 9 Gy of whole-body irradiation. Pre-treatment with cytosine arabinoside obviates the need for marrow reconstruction after irradiation. Small fragments (2–3 mm in greatest linear dimension) of tumour HXK4 (Nowak *et al.*, 1978) were implanted s.c. in each flank of the mice. Tumour HXK4 was originally propagated from a moderately differentiated carcinoma of the rectosigmoid junction.

From the 20th day after implantation, tumours were measured every 1–2 days. The largest and smallest superficial diameters were recorded and the tumour volume was calculated as (mean diameter)$^3\pi/6$. The daily volume of each tumour ($V_t$) was divided by the volume of that tumour on the first day of measurement ($V_0$) to obtain the relative tumour volume. The mean and standard error of this quotient was then plotted as a function of time after the first measurement for each group of tumours. The relative volume was calculated because inter-tumour variation in this parameter arises only during the period of measurement. The control group consisted of 50 xenografts, and each experimental group consisted of 10. Each group was measured for 5 days and the statistical significance of differences between the relative volumes of various groups of xenografts was assessed using the Mann–Whitney non-parametric test for ranked observations (Sokal & Rohlf, 1969). Experimental groups of mice were injected every 12 h with either 16,16-dimethyl PG $E_2$ methyl ester (250 $\mu$g/kg), 16,16-dimethyl PG $F_{2\alpha}$ methyl ester (250 $\mu$g/kg) or flurbiprofen (1 mg/kg).

### RESULTS

### *Mitotic rate in rat*

The doses of PG analogues and flurbiprofen were generally well tolerated by the rats, although all PG-treated animals developed mild diarrhoea. In the flurbiprofen-treated animals, there was no microscopical or histological evidence of mucosal ulceration during the 4 h period of the experiment. In the jejunal crypt epithelial cell proliferation was accelerated by 16,16-dimethyl PG $F_{2\alpha}$ at each dose

TABLE.—*Mitotic rate in jejunal crypts, colonic crypts and in DMH-induced adenocarcinomas*

| Treatment | Dose/kg | Mitotic rate: mitoses/cell/h (mean ± s.e.) Jejunal crypts | Colonic crypts | Adenocarcinomas |
|---|---|---|---|---|
| Nil (Control) | | 0·035 ± 0·002 | 0·028 ± 0·004 | 0·025 ± 0·007 |
| 16,16-dimethyl PG $E_2$ | 250 μg | 0·044 ± 0·009 | 0·021 ± 0·008 | 0·018 ± 0·002 |
| | 2·5 μg | — | — | 0·033 ± 0·008 |
| | 0·025 μg | — | — | 0·022 ± 0·001 |
| 16,16-dimethyl PG $F_{2\alpha}$ | 250 μg | 0·081* ± 0·016 | 0·017 ± 0·008 | 0† ± 0·001 |
| | 2·5 μg | 0·073* ± 0·005 | — | 0† ± 0·008 |
| | 0·025 μg | 0·051* ± 0·003 | — | 0·015 ± 0·006 |
| 6-hydroxydopamine | 100 mg | 0·009* ± 0·001 | — | — |
| 16,16-dimethyl PG $F_{2\alpha}$ after 6-hydroxydopamine | 250 μg | 0·080‡ ± 0·019 | — | — |
| Flurbiprofen | 1 mg | 0·039 ± 0·010 | 0·034 ± 0·014 | 0·068* ± 0·015 |

* Significantly different ($P < 0·05$) from control value for corresponding tissue.
† Regression coefficient for mitotic index versus duration of vinblastine treatment was negative and significantly lower ($P < 0·05$) than control value.
‡ Significantly higher ($P < 0·01$) than for the corresponding tissue treated with 6-hydroxydopamine alone.

tested, in both intact and in chemically sympathectomized rats. The log-dose response was linear with a correlation coefficient of + 0·97. Neither 16,16-dimethyl PG $E_2$ nor flurbiprofen had any statistically significant influence on jejunal-crypt cell proliferation.

FIG.—Graph of log relative tumour volume *vs* time after start of treatment. Each point represents the mean of at least 10 xenografts. Bars represent s.e. * Indicate points significantly lower ($P < 0·05$) than control xenografts. Control ••••••; Flurbiprofen -------; 16,16-dimethyl PG $E_2$ ———; 16,16-dimethyl PF $F_{2\alpha}$ —•—•—.

In the colonic-crypt epithelium none of the agents significantly influenced cell proliferation. However, in colonic adenocarcinomas, 16,16-dimethyl PG $F_{2\alpha}$ strongly inhibited cell proliferation at doses of 250 and 2·5 μg/kg and flurbiprofen accelerated it. The log-dose response had a correlation coefficient of − 0·90. Again 16,16-dimethyl PG $E_2$ was without significant effect. These results are quantitated in the Table.

### *Xenograft studies*

Both 16,16-dimethyl PG $E_2$ and 16,16-dimethyl PG $F_{2\alpha}$ inhibited xenograft growth ($P < 0·05$) but the effect of the PG $F_{2\alpha}$ analogue was more prolonged than that of the PG $E_2$ analogue. Flurbiprofen had no statistically significant effect on xenograft growth ($0·2 > P > 0·1$). These results are illustrated in the Fig.

### DISCUSSION

It is clear from the foregoing results that the PG $F_{2\alpha}$ analogue, 16,16-dimethyl PG $F_{2\alpha}$ methyl ester, is able to influence cell proliferation although, as with numerous other agents, the response varies markedly from tissue to tissue. It is not possible from the present experiments to determine whether the PG analogues are acting directly on the proliferating cells or indirectly through, for example, changes in blood supply, intestinal motility or mast-cell activity.

PG $F_{2\alpha}$ has been shown to act, in some cases at least, by raising intracellular levels of cyclic guanosine monophosphate (cGMP) (Kuehl *et al.*, 1973) and 3 other agents that have been shown to stimulate the formation of cGMP, namely noradrenaline (Schultz *et al.*, 1975), acetylcholine (Goldberg *et al.*, 1973) and serotonin (Goldberg *et al.*, 1974), as well as dibutyryl cGMP itself, have been shown to accelerate jejunal-crypt cell proliferation (Tutton & Helme, 1974; Tutton, 1974, 1977; Tutton & Barkla, 1979*b*). However, in the case of colonic adenocarcinoma, whilst both serotonin (Tutton & Barkla, 1978*b*) and dibutyryl cGMP (Tutton & Barkla, 1979*b*) promote cell division, the PG $F_{2\alpha}$ analogue inhibits both cell division and xenograft growth.

PG $E_2$ has been shown to elevate the intracellular levels of cyclic adenosine monophosphate (cAMP) (Kuehl *et al.*, 1970) and the transient inhibition of xenograft growth in animals treated with the PG $E_2$ analogue resembles the influences of adrenaline, also known to raise cellular cAMP levels (Sutherland & Rall, 1960) on the tumour HXK4 in xenograft (Tutton & Steel, 1979). The influence of adrenaline was prolonged by theophylline, an agent known to inhibit the enzyme phosphodiesterase, which degrades cAMP.

When interpreting the apparent inconsistency between the influence of injected prostaglandin analogues (significantly altering cell proliferation and tumour growth) and flurbiprofen (having no effect) on the jejunal crypts and on xenografts, it must be remembered that inhibition of cyclo-oxygenase by flurbiprofen will interfere with the production of thromboxanes and prostacyclin (Johnson *et al.*, 1976) as well as PG $E_2$ and PG $F_{2\alpha}$. Alternatively, flurbiprofen may be ineffective as a cyclo-oxygenase inhibitor in the intestinal epithelium. It is difficult to investigate the influence of thromboxane $A_2$ and prostacyclin on cell division, because each of these substances is very unstable.

Various prostaglandins and their synthetic analogues have been shown to influence cell proliferation in other neoplastic and non-neoplastic tissues. Kurland & Moore (1977) reported stimulation of haemopoietic stem cells by PG $E_2$, whilst inhibition of cell proliferation in such tumours as B16 melanoma (Santoro *et al.*, 1977) and plasmacytoma (Naseem & Hollander, 1973) has also been reported.

This work was done during the tenure of a research grant awarded by the Anti-Cancer Council of Victoria. The authors wish to thank Professor D. M. de Kretser, Dr J. L. Millar and Dr G. G. Steel for their encouragement in this project, and Fiona Christensen and Jill Riddiford for their skilled technical assistance. Prostaglandin analogues used in this work were the generous gift of the Upjohn Company and flurbiprofen was generously donated by the Boots Company (Australia) Pty Ltd.

### REFERENCES

BLISS, C. I. (1967) *Statistics in Biology*, Vol. 1. New York: McGraw-Hill. p. 420.

CROOK, D. & COLLINS, A. J. (1975). Prostaglandin synthetase activity from human rheumatoid synovial tissue and its inhibition by non-steroidal anti-inflammatory drugs. *Prostaglandins*, **9**, 857.

DRUCKREY, H., PREUSSMAN, R., MATZKIES, F. & IVANKOVIC, S. (1967) Selektive Erzeugung von Darmkrebs bei Ratten durch 1,2-Diemethylhydrazin. *Naturwissenschaften*, **54**, 285.

GOLDBERG, N. D., HADDOX, M. K., DUNHAM, E., LOPEZ, C. & HADDEN, J. W. (1974) In *Control of Proliferation in Animal Cells*. Ed. Clarkson & Baserga. New York: Cold Spring Harbor Press. p. 609.

GOLDBERG, N. D., HADDOX, M. K., HARTLE, D. K. & HADDEN, J. W. (1973) Pharmacology and the future of man. *Proc. Vth Int. Cong. Pharmacol.* Basel: Karger. p. 146.

JOHNSON, R. A., MORTON, D. R., KINNER, J. H. & 8 others (1976) The chemical structure of prostaglandin X (prostacyclin). *Prostaglandins*, **12**, 915.

KADOWITZ, P. J., SWEET, C. S. & BRODY, M. J. (1972) Enhancement of sympathetic neutrotransmission by prostaglandin $F_{2\alpha}$ in the cutaneous vascular bed of dog. *Eur. J. Pharmacol.*, **18**, 189.

KUEHL, F. A., HUMES, J. L., TARNOFF, J., CIRILLO, V. J. & HAM, E. A. (1970) Prostaglandin receptor site: Evidence for an essential role in the action of luteinizing hormone. *Science*, **169**, 883.

KUEHL, F. A., CIRILLO, V. J., HAM, E. A. & HUMES, J. L. (1973) The regulatory role of the prostaglandins on the cyclic AMP system. In *Advances in*

*Biosciences*. Ed Bergstrom & Bernhard. Oxford: Pergamon Press. p. 155.

KURLAND, J. I. & MOORE, M. A. S. (1977) Modulation of haemopoiesis by prostaglandins. *Exp. Hematol.*, **5**, 357.

NASEEM, S. M. & HOLLANDER, V. P. (1973) Insulin reversal of growth inhibition of plasma cell tumour by prostaglandins or adenosine-3′,5′-monophosphate. *Cancer Res.*, **33**, 2909.

NOWAK, K., PECKHAM, M. J. & STEEL, G. G. (1978) Variations in the response of xenografts of colorectal carcinoma to chemotherapy. *Br. J. Cancer*, **37**, 576.

SANTORO, M. G., PHILPOTT, G. W. & JAFFE, B. M. (1977) Dose dependent inhibition of B-16 melanoma *in vivo* by a synthetic analogue of PG $E_2$. *Prostaglandins*, **14**, 645.

SHULTZ, G., SHULTZ, K. & HARDMAN, J. G. (1975) Effects of norepinephrine on cyclic nucleotide levels in the ductus deferens of the rat. *Metabolism*, **24**, 429.

SOKAL, R. R. & ROHLF, F. J. (1969) *Biometry*. San Francisco: W. H. Freeman & Co. p. 392.

STEEL, G. G., COURTENAY, V. D. & ROSTOM, A. Y. (1978) Improved immune-suppression techniques for xenografting human tumours. *Br. J. Cancer*, **37**, 261.

SUTHERLAND, E. W. & RALL, T. W. (1960) The relation of adenosine-3′,5′-phosphate and phosphorylase to the action of catecholamines and other hormones. *Pharmacol. Rev.*, **12**, 265.

TUTTON, P. J. M. (1974) The influence of serotonin on crypt cell proliferation in the jejunum of rat. *Virchows Arch.* [*Cell Pathol.*], **16**, 79.

TUTTON, P. J. M. (1976) The influence of histamine on epithelial cell proliferation in the jejunum of rat. *Clin. Exp. Pharmacol. Physiol.*, **3**, 369.

TUTTON, P. J. M. (1977) Neural and endocrine control systems acting on the population kinetics of the intestinal epithelium. *Med. Biol.*, **55**, 201.

TUTTON, P. J. M. & BARKLA, D. H. (1976) Cell proliferation in the descending colon of dimethylhydrazine treated rats and in dimethylhydrazine induced adenocarcinomata. *Virchows Arch.* [*Cell Pathol.*], **21**, 147.

TUTTON, P. J. M. & BARKLA, D. H. (1977) The influence of adrenoceptor activity on cell proliferation in colonic crypt epithelium and in colonic adenocarcinomata. *Virchows Arch.* [*Cell Path.*], **24**, 139.

TUTTON, P. J. M. & BARKLA, D. H. (1978*a*) Stimulation of cell proliferation by histamine $H_2$-receptors in dimethylhydrazine-induced adenocarcinomata. *Cell Biol. Int. Rep.*, **2**, 199.

TUTTON, P. J. M. & BARKLA, D. H. (1978*b*) The influence of serotonin on the mitotic rate in the colonic crypt epithelium and in colonic carcinoma in rats. *Clin. Exp. Pharmacol. Physiol.*, **5**, 91.

TUTTON, P. J. M. & BARKLA, D. H. (1979*a*) Neural control of colonic epithelial cell proliferation. *Cancer* (In press).

TUTTON, P. J. M. & BARKLA, D. H. (1979*b*) A final common pathway promoting cell proliferation in normal and neoplastic intestinal epithelia. *Proc. Conf. Cell Proliferation Gastrointestinal Tract.* (In press).

TUTTON, P. J. M. & HELME, R. D. (1974) The influence of adrenoceptor activity on crypt cell proliferation in the rat jejunum. *Cell Tiss. Kinet.*, **7**, 125.

TUTTON, P. J. M. & STEEL, G. G. (1979) Influence of biogenic amines on the growth of xenografted human colorectal carcinomas. *Br. J. Cancer*, **40**, 743.